# Investigation of Oral Shedding of Torquetenovirus (TTV) in Moderate-to-Severe COVID-19 Hospitalised Patients

**DOI:** 10.3390/v16060831

**Published:** 2024-05-24

**Authors:** Rafael Antônio Velôso Caixeta, Alexandre Mendes Batista, Matheus Willian Caetano, Michelle Palmieri, Gabriela Schwab, Rodrigo Melim Zerbinati, Andressa Silva Pereira Victor, Camila de Barros Gallo, Tânia Regina Tozetto-Mendoza, Roger Junges, Karem L. Ortega, André Luiz Ferreira Costa, Dmitry José de Santana Sarmento, Débora Pallos, José Angelo Lauletta Lindoso, Simone Giannecchini, Paulo Henrique Braz-Silva

**Affiliations:** 1 Department of Stomatology, School of Dentistry, University of São Paulo, São Paulo 05508-000, Brazil; rafaelcaixeta@usp.br (R.A.V.C.); matheuswillian@usp.br (M.W.C.); mimyselfpalm@yahoo.com.br (M.P.); camilagallo@usp.br (C.d.B.G.); klortega@usp.br (K.L.O.); 2 Laboratory of Virology (LIM-52-HCFMUSP), Institute of Tropical Medicine, University of São Paulo School of Medicine, São Paulo 05403-000, Brazil; a.batista@usp.br (A.M.B.); gabriela.schwab@usp.br (G.S.); rmzerbinati@usp.br (R.M.Z.); andressa.silvapvictor@gmail.com (A.S.P.V.); tozetto@usp.br (T.R.T.-M.); 3 Institute of Oral Biology, Faculty of Dentistry, University of Oslo, 0313 Oslo, Norway; roger.junges@odont.uio.no; 4 Oral Medicine, Oral Surgery and Implantology Unit (MedOralRes), Faculty of Medicine and Dentistry, University of Santiago de Compostela, 15705 Santiago de Compostela, Spain; 5 Post-Graduate Program in Dentistry, Cruzeiro do Sul University, São Paulo 08060-070, Brazil; alfcosta@gmail.com; 6 School of Dentistry, State University of Paraíba, Araruna 58233-000, Brazil; dmitry.sarmento@gmail.com; 7 School of Dentistry, University of Santo Amaro, São Paulo 04743-030, Brazil; dpallos@prof.unisa.br; 8 Emílio Ribas Institute of Infectious Diseases, São Paulo 01246-900, Brazil; jlindoso@usp.br; 9 Laboratory of Protozoology (LIM-49-HC-FMUSP), Institute of Tropical Medicine, University of São Paulo School of Medicine, São Paulo 01246-903, Brazil; 10 Department of Infectious Diseases, University of São Paulo School of Medicine, São Paulo 01246-903, Brazil; 11 Department of Experimental and Clinical Medicine, University of Florence, 50134 Florence, Italy

**Keywords:** biomarkers, saliva, torquetenovirus, SARS-CoV-2, COVID-19

## Abstract

Background. Torquetenovirus (TTV) is a small DNA virus constituting the human virome. High levels of TTV-DNA have been shown to be associated with immunosuppression and inflammatory chronic disorders. Aim. To assess the possible association between the salivary viral load of TTV-DNA in patients hospitalised due to COVID-19 and disease severity. Methods. Saliva samples collected from 176 patients infected with SARS-CoV-2 were used to investigate the presence of SARS-CoV-2 and TTV-DNA by use of real-time RT-PCR. Results. The majority of patients were male with severe COVID-19. Presence of SARS-CoV-2 was observed in the saliva of 64.77% of patients, showing TTV-DNA in 55.68% of them. Patients with impaired clinical conditions (*p* < 0.001), which evolved to death (*p* = 0.003), showed a higher prevalence of TTV-DNA. The median viral load in patients with severe condition was 4.99 log_10_ copies/mL, in which those who were discharged and those evolving to death had values of 3.96 log_10_ copies/mL and 6.27 log_10_ copies/mL, respectively. A statistically significant association was found between the distribution of TTV-DNA viral load in saliva samples and severity of COVID-19 (*p* = 0.004) and disease outcomes (*p* < 0.001). Conclusions. These results indicate that TTV-DNA in saliva could be a useful biomarker of COVID-19 severity and prognosis.

## 1. Introduction

Torquetenovirus (TTV) is a small, non-enveloped, singled-stranded DNA virus belonging to the family of *Anelloviridae*. This virus species was initially discovered in 1997 as a possible causative agent of a case of post-transfusion hepatitis in humans [1]. It is currently known that these viruses infect humans at high rates, reaching approximately 90 percent of the population, but are not associated with any specific human disease [2,3]. Lymphocyte T cells are one of the main sites of TTV replication, but other cells and tissues can be targeted by these viruses, such as bone marrow, the airways, and the liver [4,5].

In the past decades, the use of metagenomic sequencing has allowed the discovery that TTV makes up of more than 65 percent of the virome in post-transplantation patients, whereas only 10 percent in non-immunosuppressed individuals [2,6,7]. TTV DNAemia is documented in healthy individuals and is relatively stable regarding its magnitude over the years [8]. However, TTV DNAemia levels have shown oscillations in immunosuppressed patients, meaning that high loads of TTV DNA have a clear, consistent, and direct association with the intensity of individual immune functionality [9,10,11,12]. In addition to immunosuppression conditions, high plasma loads of TTV-DNA were observed in patients with inflammatory, chronic disorders compared to healthy individuals, as in the cases of asthma, chronic bronchiectasis, and chronic obstructive pulmonary disease [13,14]. Thus, immunological disorders and anti-inflammatory or immunosuppressant medications can affect the immune balance and, consequently, favour TTV replication [4,15,16]. This allows the identification of high levels of TTV viral load and their detection in different samples, such as plasma, saliva, and serum [15,17]. In this context, Coronavirus Disease 19 (COVID-19) has been abundantly associated with a dysregulation of the pro-inflammatory milieu and immunopathogenic characteristics observed in moderate-to-severe cases, which leads to an impairment of acute respiratory discomfort syndrome and, consequently, multiple organ failure and death [18,19]. Therefore, the possibility that TTV replication is influenced by COVID-19 immunological dysregulation points to the hypothesis that detection of TTV-DNA can play a role as a severity biomarker for monitoring moderate-to-severe cases of COVID-19. Thus, several studies have investigated TTV viral load in plasma and saliva samples, regardless of SARS-CoV-2 status and COVID-19 severity [20,21]. In this context, saliva has several advantages and applicability as a potential biomarker for the diagnosis and monitoring of SARS-CoV-2 [20]. The aim of this present study was to assess TTV-DNA viral load in the saliva of patients diagnosed with COVID-19 who were hospitalised due to complications and to investigate the existence of an association of TTV status with level of severity of the disease and outcomes.

## 2. Materials and Methods

### 2.1. Ethical Aspects

This study was approved by the Research Ethics Committee of the Emilio Ribas Institute of Infectology, under protocol numbers CAAE 35589320.6.0000.0061 and 4205770 (August/2020). This study was also carried out according to the ethical guidelines set by institutional and national committees, and in accordance with the 1964 Helsinki Declaration and later amendments.

### 2.2. Patients and Samples

The enrolled study population was a convenience sample and consisted of adult male and female patients with clinical manifestations and a molecular diagnosis of COVID-19, who were either in the infirmary ward (moderate disease) or the intensive care unit (severe and critical disease) of the Emilio Ribas Institute of Infectology, between October 2021 and December 2021. The enrolled criteria were that patients had a positive SARS-CoV-2 diagnosis confirmed by molecular real-time RT–PCR analysis on nasopharyngeal swab samples collected in the initial evaluation. The patients included in this study signed an informed consent form and were then distributed according to their clinical condition: patients with moderate disease (characterised by the presence of influenza symptoms associated with lung impairment < 50%, measured by computed tomography, and O_2_ saturation > 93% in room air, thus requiring admission to the infirmary ward) and patients with severe and critical disease (characterised by a respiratory rate > 30 breaths per minute, O_2_ saturation < 93% in room air and lung impairment > 50%, measured by computed tomography, requiring admission to intensive care unit (ICU)). The clinical and demographic data of patients were obtained from medical records (Table 1). The main exclusion criteria were patients with asymptomatic to no limitation activity COVID-19 condition or those who were pregnant and below 18 years old.

### 2.3. Saliva Sample Collection

Saliva samples were collected on the day of admittance of patients who were hospitalised due to COVID-19 complications. The average time from the onset of symptoms to the day of sampling was 7.14 days. Saliva collection was performed using a Salivette^®^ collection kit (Sarstedt AD & Co., Ltd., Nuembrecht, Germany) and the samples collected were labelled accordingly. The patients were instructed to brush their teeth until 2 h before saliva collection (i.e., between 7:00 a.m. and 11:00 a.m.) and to not ingest food or beverages. Instructions on saliva collection were the following: after removing the cotton roll from the collection tube, the individual was asked to chew the cotton roll for approximately 90 s until it was soaked with saliva and then to place it into the collection tube. Next, the tube containing the soaked cotton was centrifuged at 1000× *g* for 2 min at room temperature so that the saliva was deposited at the bottom of the tube. Lastly, the cotton roll was removed and the tube with the saliva sample was closed, before being stored in a freezer at −80 °C. In the case of patients on mechanical ventilation, the dentist surgeon kept the cotton roll inside the patient’s oral cavity using tweezers for 90 s before following the above-described steps.

### 2.4. Molecular Analyses

#### 2.4.1. Detection of SARS-CoV-2

Total nucleic acid was extracted and purified using virus RNA/DNA extraction kit (Extracta, Loccus, Cotia, Brazil) and an Extracta 32 DNA/RNA extraction machine (Extract, Loccus, Brazil) according to the manufacturer’s instructions, in which 200 μL of saliva of each sample was used for a final elution of 100 μL. The viral RNA of SARS-CoV-2 was detected using the SARS-CoV-2 RT-qPCR Reagent kit (PerkinElmer, Turku, Finland), according to the manufacturer’s instructions. The protocol was aimed at genes ORF1ab and nucleocapsid protein (N), including the use of an internal control. In summary, 14 μL of extracted RNA was added to a previously prepared mixture of reagents containing 1 μL of CoV2 Reagent A and 5 μL of CoV2 Enzyme Mix, totalling a final volume of 20 μL of reaction. The protocol proceeded with 2-min cycling at 25 °C and 15-min cycling at 50 °C for reverse transcription, followed by an initial step of 2-min denaturation at 95 °C and 45 amplification cycles at 95 °C for 3 s and at 60 °C for 30 s.

#### 2.4.2. Detection and Quantification of TTV

TTV-DNA in saliva was detected by means of a real-time-polymerase chain reaction (qPCR) and using probes and primers specific for the untranslated region (UTR), which is a highly conserved region of the genome. A forward primer (5′-GTGCCGIAGGTGAGTTTA-3′), reverse primer (5′-AGCCCGGCCAGTCC-3′), and probe (FAM5′-TCAAGGGGCAATTCGGGCT-3′MGBNFQ) were used, as described by Maggi et al., (2005) elsewhere [22]. A standard curve for known amounts of synthetic oligonucleotide and a TaqMan qPCR kit were used to obtain an absolute quantification of TTV-DNA, as described by Tozetto-Mendoza et al. (2020). TTV-DNA amplification was performed according to the protocols established for the TaqMan Universal PCR master mix kit (Thermo Fisher Scientific, Warrington, UK). Data analysis was performed using the QuantStudio Design & Analysis software, version 1.4.1. With regard to both positive and negative controls for TTV, saliva samples stored in the repository of the Institute of Tropical Medicine Laboratory of Virology at the University of São Paulo School of Medicine, Brazil were used. The detection range of salivary TTV was between 1.6 and 7.4 log_10_ copies/mL, meaning that the lowest limit of detection for TTV was 40 copies/mL (1.6 log_10_ copies/mL) [23].

### 2.5. Statistical Analysis

The resulting data were analysed using SPSS software, version 20.0 (SPSS, Inc., Chicago, IL, USA). A data normality test showed a normal distribution for age (Shapiro-Wilk’s test, *p* = 0.065) and an abnormal one for TTV quantification (Shapiro-Wilk’s test, *p* < 0.001), meaning that the Student’s *t*-test was used to assess the association between age and COVID-19 severity, whereas the Mann-Whitney’s test was used to assess the association between outcome and COVID-19 severity. The Pearson’s chi-square test or Fisher’s exact test was used for comparison of categorical variables in relation to TTV positivity and COVID-19 severity. All statistical tests were performed at a significance level of 5%.

## 3. Results

In this study, we included 176 patients, who were all cases positive for SARS-CoV-2 as determined by a nasopharyngeal swab test (Table 1). The study population was distributed according to inpatient ward and severity of COVID-19, in which 88 patients were classified as moderate cases and then admitted to the infirmary ward, whereas the other 88 patients were classified as severe cases and admitted to the ICU. Table 1 reports the demographic and clinical characteristics of patients with signs, symptoms, and outcomes of disease. Most of the patients were male, who presented more severe conditions compared to women (Pearson’s chi-square test; *p* = 0.014). The mean age of the study population was 53.89 ± 13.80 years old, which was found to be similar between moderate (53.82 ± 14.43) and severe (53.97 ± 13.23) cases (Student’s *t*-test; *p* = 0.944). Among the saliva samples examined, 64.8% were positive for SARS-CoV-2, with a significant positivity difference (*p* < 0.05) between samples collected from severe COVID-19 cases (72.7%) compared to moderate cases (56.8%). With regard to the parameters “need of oxygen support”, “orotracheal intubation”, “responsiveness” (ability to respond to verbal stimuli), “mobility” (ability to move around the ward), “oral feeding”, and “use of nasogastric tube”, the worst scenarios were observed in severe patients compared to those in the infirmary ward, with all results being statistically significant. The majority of hospitalised patients (88.1%) had never been vaccinated against COVID-19. Proportionally, the prevalence of death was higher among severe patients. With regard to the signs and symptoms of patients evaluated in this present study, there was a similarity in their distribution between moderate and severe cases (Table 1).

First, the association of the presence of TTV-DNA in the saliva samples of the two groups of study patients (COVID-19 moderate cases and COVID-19 severe cases, respectively) was investigated with clinical and demographic parameters. Among the 176 saliva samples examined, 98 (55.7%) were TTV-DNA positive. The prevalence of TTV-DNA was higher in male individuals and in more severe cases, but no statistical significance was observed regarding TTV status in saliva samples between male and female patients and between moderate and severe cases. No association was found between the presence of SARS-CoV-2 positivity and TTV status in saliva. Intubated, unresponsive patients without mobility who were on a nasogastric tube or could not be orally fed had a higher prevalence of TTV, with all results being statistically significant, except for the variable “mobility”. Patients who evolved to death had the highest prevalence of TTV-DNA compared to those who were discharged. Overall, variables indicating severity of disease showed an association with positivity for TTV-DNA (Table 2).

Finally, Table 3 reports the distribution of TTV viral load in saliva samples in relation to the rank of COVID-19 severity and outcomes, with both associations being statistically significant. The median TTV-DNA viral load in patients with severe condition was 4.99 log10 copies/mL, exhibiting values of 3.96 log10 copies/mL and 6.27 log10 copies/mL in those being discharged and in those evolving to death, respectively. Overall, the highest values of severity were found in severe patients and in those who evolved to death. In this case, ranks (order) were assigned for each observation, from 1 to 176, which was the total size of the combined samples. When calculating the TTV ranks, it was observed that the TTV values were in fact higher in patients with severe disease and who died, compared to patients with moderate disease and who were discharged, respectively.

## 4. Discussion

In this study, TTV-DNA viral status in the saliva of 176 SARS-CoV-2 infected hospitalised patients was investigated, and its relationship with the severity of COVID-19 outcomes was assessed. All the patients included in our study had a diagnosis of COVID-19 performed by means of molecular detection of SARS-CoV-2 in nasopharyngeal swab samples, whereas the salivary presence of the virus was detected in 64.8% of subjects, showing the persistence of the virus in this fluid. This confirms the relevance of saliva sampling as a fluid with diagnostic and prognostic potential to allow for the follow-up of patients hospitalised due to COVID-19 complications, not only by detecting the virus, but also as other a biomarker of infection [20,21,24,25]. Among all patients examined in this present study, the majority of moderate or severe cases of COVID-19 involved male patients (*p* = 0.014). As studies show the same male predominance [8,26,27,28], some hypotheses have been raised in an attempt to explain such prevalence, including mechanisms of cell protection mediated by the presence of female hormones, possible gender-related differences in immune responses, and even behavioural differences between men (e.g., smoking, which is more prevalent in male individuals) and women [29,30,31]. The higher prevalence of TTV-DNA in male individuals and in more severe cases is similar to the findings reported by other studies of individuals with different causes of immunosuppression [32,33,34]. No significant difference was observed in SARS-CoV-2 detected in the saliva (52.6%) of TTV-positive patients compared to negative ones (47.4%).

The association between TTV viraemia and COVID-19 severity was assessed subgrouping patients in 88 moderate subjects (classified as moderate cases and then admitted to the infirmary ward) and in 88 severe subjects (classified as severe cases and admitted to the ICU). With regard to the clinical parameters of COVID-19 indicating greater severity of the disease, this present study assessed inpatient environment (i.e., infirmary or ICU), need of oxygen support, orotracheal intubation, responsiveness, mobility, oral feeding, and use of a nasogastric tube similarly to other studies [11,34,35]. In particular, TTV-DNA in the saliva of patients with moderate-to-severe conditions of COVID-19 showed a prevalence of 50% and 61.4% in moderate and severe patients, respectively. Quantitatively, it was observed that a higher prevalence and viral load of TTV was detected in severe ICU patients who evolved to death compared to moderate ones (*p* < 0.001), suggesting an association between the presence of TTV-DNA and a worse prognosis of the disease. Overall, TTV can be detected using different biological samples [34] and its observed prevalence can vary according to individual conditions. Our data support those in the literature reporting high TTV prevalence detected in saliva [30,35,36,37,38]. For instance, the prevalence of TTV-DNA in the plasma, swab, and saliva of healthy individuals are 65% [8], 71.8% [39], and 85%, respectively [27]. Therefore, the difference in the behaviour of TTV regarding different biological compartments might explain the differences observed in the literature.

The detection of TTV-DNA viral loads depends on the condition, immunosuppression levels, and the population studied [2,10,12]. When considering the immunopathogenesis and inflammatory aspects of COVID-19, it is plausible to assume that TTV viraemia increases, suggesting its role as a possible biomarker of severity [11,22,40,41,42]. However, investigation has also pointed out that, although TTV-DNA load is useful for mortality risk assessment in COVID-19 patients, it is a poor surrogate marker of inflammation [11]. Possible explanations for such results could be related to different methodology and samples used (plasma versus saliva samples), the presence of a different nature of the TTV’s genogroup, and its ability to stimulate inflammatory responses, thus leading to pro-inflammatory conditions [41]. The higher viral load of TTV observed in our study can be explained by considering the possibility that TTV replication can be stimulated by a pro-inflammatory environment, as well as by immunopathogenic characteristics observed in moderate-to severe cases of COVID-19, such as lymphopenia and pro-inflammatory cytokine storms, with the latter worsening acute respiratory discomfort syndrome and damaging tissues, which can evolve to death [18,19]. These findings are corroborated by the kinetics observed in the study by Mendes-Correa et al. (2021), in which TTV in the saliva and nasopharyngeal swabs of SARS-CoV-2-positive patients was evaluated. The authors reported that the levels of TTV in these patients reduced significantly as respiratory symptoms improved and the levels of SARS-CoV-2 decreased [21]. However, the role of the presence of different genogroups of TTV should be also investigated. In the case of SARS-CoV-2-positive patients with moderate condition, decreased TTV viral load and respiratory symptoms are reported [21]. Conversely, in critical patients hospitalised due to COVID-19, a TTV median of 2.8 log_10_ copies/mL was observed at admission, achieving a peak of 4.75 log_10_ copies/mL in the fourth week [11]. Additionally, in another study, the prevalence and mean viral load of TTV observed in SARS-CoV-2-positive and negative patients were not significantly different [38]. In our study, we found that disease severity, outcomes, and salivary TTV viral load were statistically associated as severe patients and those who evolved to death had higher median TTV level values. Respective ranks were 99.11 and 129.45 compared to those of moderate patients (77.89) and discharged ones (82.95).

Several studies have tried to establish minimum and maximum limits for detection of TTV in order to predict the risks and outcomes of an immunosuppression condition in an individual reporting that viral load values above 9.5 log_10_ copies/mL reflect a critical condition of the patient, as high immunosuppression is associated with higher risks of infection [9,43], whereas values below 7.0 log10 copies/mL are related to risk of post-transplantation rejection due to very low immunosuppression [44]. In the case of COVID-19 patients, there is no such a limit, but the presence of detectable TTV viral loads (i.e., ≥3.3 log10 copies/mL) in the plasma of severe patients has been associated with higher risks of the development of secondary infections, and viral loads increasing in a median of 25.8 log 10 copies/day/mL is related to a higher risk of death [11]. The median TTV viral load (copies/mL) of moderate and severe patients was 3.04 log10 and 4.99 log10, respectively. Regarding outcomes, the TTV viral load of patients who were discharged was 3.96 log10 and of those who evolved to death was 6.27 log10. These findings may imply a perspective regarding the use of TTV salivary viral load detection to monitor COVID-19 hospitalised patients.

The main limitations of this study include the following: The small size of the study population, also lacking patients with mild COVID-19 symptoms, and the absence of investigations to compare the level of immunosuppression, such as lymphocyte counts and the presence of opportunistic infections, as observed elsewhere [11,39]. Additionally, TTV viral load was not evaluated in samples other than saliva, meaning that it was not possible to elucidate possible differences in the behaviour of different biological compartments. Finally, TTV genogrouping was not carried out to investigate its potential role in severe COVID-19 outcomes.

Overall, the results presented in this study suggest a potential direct association between the presence of different viral load levels of TTV and severity and outcomes in COVID-19 hospitalised patients. Thus, additional investigation will be useful to confirm TTV as a biomarker of severity and prognosis of the disease.

## Figures and Tables

**Table 1:** Demographic and clinical characteristics of study patients.

Variable	COVID-19 Moderate (n 88)	COVID-19 Severe (n 88)	Total (n 176)	*p*
**Gender**				
Male n (%)	45 (51.1)	61 (69.3)	106 (60.2)	0.014 *^(1)^
Female n (%)	43 (48.9)	27 (30.7)	70 (39.8)	
Age (mean ± SD years)	53.82 ± 14.43	53.97 ± 13.23	53.89 ± 13.80	0.944
**Saliva—SARS-CoV-2**				
Negative n (%)	38 (43.2)	24 (27.3)	62 (35.2)	0.027 *^(1)^
Positive n (%)	50 (56.8)	64 (72.7)	114 (64.8)	
**Breathing in room air spontaneous**				
No n (%)	68 (77.3)	69 (78.4)	137 (77.8)	0.856 ^(1)^
Yes n (%)	20 (22.7)	19 (21.6)	39 (22.2)	
**Oxygen support**				
No n (%)	26 (29.5)	45 (51.1)	71 (40.3)	<0.004 *^(1)^
Yes n (%)	62 (70.5)	43 (48.9)	105 (59.7)	
**Orotracheal intubation (OTI)**				
No n (%)	82 (93.2)	63 (71.6)	145 (82.4)	<0.001 *^(1)^
Yes n (%)	6 (6.8)	25 (28.4)	31 (17.6)	
**Responsiveness**				
No n (%)	9 (10.2)	26 (29.5)	35 (19.9)	0.001 *^(1)^
Yes n (%)	79 (89.8)	62 (70.5)	141 (80.1)	
**Mobility**				
No n (%)	23 (26.1)	61 (69.3)	84 (47.7)	<0.001 *^(1)^
Yes n (%)	65 (73.9)	27 (30.7)	92 (52.3)	
**Oral feeding**				
No n (%)	7 (8.0)	27 (30.7)	34 (19.3)	<0.001 *^(1)^
Yes n (%)	81 (92.0)	61 (69.3)	142 (80.7)	
**Use of nasogastric tube**				
No n (%)	81 (92.0)	63 (71.6)	144 (81.8)	<0.001 *^(1)^
Yes n (%)	7 (8.0)	25 (28.4)	32 (18.2)	
**History of COVID-19 vaccination**				
No n (%)	77 (87.5)	78 (88.6)	155 (88.1)	0.816 ^(1)^
Yes n (%)	11 (12.5)	10 (11.4)	21 (11.9)	
**Outcome**				
Discharge n (%)	84 (95.5)	71 (80.7)	155 (88.1)	0.003 ^(1)^
Death n (%)	4 (4.5)	17 (19.3)	21 (11.9)	
**Signs & Symptoms**				
**Fever**				
No n (%)	33 (37.5)	30 (34.1)	63 (35.8)	0.637 ^(1)^
Yes n (%)	55 (62.5)	58 (65.9)	113 (64.2)	
**Cough**				
No n (%)	27 (30.7)	29 (33.0)	56 (31.8)	0.746 ^(1)^
Yes n (%)	61 (69.3)	59 (67.0)	120 (68.2)	
**Headache**				
No n (%)	58 (65.9)	55 (62.5)	113 (64.2)	0.637 ^(1)^
Yes n (%)	30 (34.1)	33 (37.5)	63 (35.8)	
**Sore throat**				
No n (%)	70 (79.5)	74 (84.1)	144 (81.8)	0.434 ^(1)^
Yes n (%)	18 (20.5)	14 (15.9)	32 (18.2)	
**Myalgia**				
No n (%)	55 (62.5)	54 (61.4)	109 (61.9)	0.877 ^(1)^
Yes n (%)	33 (37.5)	34 (38.6)	67 (38.1)	
**Fatigue**				
No n (%)	46 (52.3)	50 (56.8)	96 (54.5)	0.545 ^(1)^
Yes n (%)	42 (47.7)	38 (43.2)	80 (45.5)	
**Coryza**				
No n (%)	78 (88.6)	71 (80.7)	149 (84.7)	0.143 ^(1)^
Yes n (%)	10 (11.4)	17 (19.3)	27 (15.3)	
**Dyspnoea**				
No n (%)	33 (37.5)	35 (39.8)	68 (38.6)	0.757 ^(1)^
Yes n (%)	55 (62.5)	53 (60.2)	108 (61.4)	
**Anosmia/Ageusia**				
No n (%)	69 (78.4)	59 (67.0)	128 (72.7)	0.091 ^(1)^
Yes n (%)	19 (21.6)	29 (33.0)	48 (27.3)	
**Nausea/Vomit**				
No n (%)	79 (89.8)	77 (87.5)	156 (88.6)	0.635 ^(1)^
Yes n (%)	9 (10.2)	11 (12.5)	20 (11.4)	
**Diarrhoea**				
No n (%)	72 (81.8)	76 (86.4)	148 (84.)	0.410 ^(1)^
Yes n (%)	16 (18.2)	12 (13.6)	28 (15.9)	
**Inappetence**				
No n (%)	78 (88.6)	81 (92.0)	159 (90.3)	0.444 ^(1)^
Yes n (%)	10 (11.4)	7 (8.0)	17 (9.7)	
**Facial pain**				
No n (%)	86 (97.7)	83 (94.3)	169 (96.0)	0.444 ^(2)^
Yes n (%)	2 (2.3)	5 (5.7)	7 (4.0)	
**Dizziness**				
No n (%)	82 (93.2)	86 (97.7)	168 (95.5)	0.278 ^(2)^
Yes n (%)	6 (6.8)	2 (2.3)	8 (4.5)	
**Abdominal pain**				
No n (%)	87 (98.9)	87 (98.9)	174 (98.9)	0.999 ^(2)^
Yes n (%)	1 (1.1)	1 (1.1)	2 (1.1)	

^(1)^ Pearson’s chi-square test; ^(2)^ Fisher’s exact test; * statistically significant (*p* < 0.05).

**Table 2:** Relationship between positivity for TTV DNA status and study variables.

Variable	TTV DNA Status			*p*
	Negative (n. 78)	Positive (n. 98)	Total (n. 176)	
**Gender**				
Male n (%)	42 (39.6)	64 (60.4)	106 (100)	0.123 ^(1)^
Female n (%)	36 (51.4)	34 (48.6)	70 (100)	
**Disease severity**				
Moderate n (%)	44 (50.0)	44 (50.0)	88 (100)	0.129 ^(1)^
Severe n (%)	34 (38.6)	54 (61.4)	88 (100)	
**Saliva—SARS-CoV-2**				
Negative n (%)	24 (38.7)	38 (61.3)	62 (100)	0.269 ^(1)^
Positive n (%)	54 (47.4)	60 (52.6)	114 (100)	
**Breath in room air spontaneous**				
No n (%)	61 (44.5)	76 (55.5)	137 (100)	0.917 ^(1)^
Yes n (%)	17 (43.6)	22 (56.4)	39 (100)	
**Oxygen support**				
No n (%)	24 (33.8)	47 (66.2)	71 (100)	0.021 *^(1)^
Yes n (%)	54 (51.4)	51 (48.6)	105 (100)	
**Orotracheal intubation (OTI)**				
No n (%)	71 (49.0)	74 (51.0)	145 (100)	0.007 *^(1)^
Yes n (%)	7 (22.6)	24 (77.4)	31 (100)	
**Responsiveness**				
No n (%)	10 (28.6)	25 (71.4)	35 (100)	0.036 *^(1)^
Yes n (%)	68 (48.2)	73 (51.8)	141 (100)	
**Mobility**				
No n (%)	32 (38.1)	52 (61.9)	84 (100)	0.112 ^(1)^
Yes n (%)	46 (50.0)	46 (50.0)	92 (100)	
**Oral feeding**				
No n (%)	8 (23.5)	26 (76.5)	34 (100)	0.007 *^(1)^
Yes n (%)	70 (49.3)	72 (50.7)	142 (100)	
**Use of nasogastric tube**				
No n (%)	71 (49.3)	73 (50.7)	144 (100)	0.005 *^(1)^
Yes n (%)	7 (21.9)	25 (78.1)	32 (100)	
**History of COVID-19 vaccination**				
No n (%)	68 (43.9)	87 (56.1)	155 (100)	0.746 ^(1)^
Yes n (%)	10 (47.6)	11 (52.4)	21 (100)	
**Outcome**				
Discharge n (%)	75 (48.4)	80 (51.6)	155 (100)	0.003 *^(1)^
Death n (%)	3 (14.3)	18 (85.7)	21 (100)	

^(1)^ Pearson’s chi-square test; * statistically significant (*p* < 0.05).

**Table 3:** Distribution of the quantification of TTV DNA in saliva (log_10_ copies/mL) in relation to the rank of severity.

Variable	N	Median	IQR	Rank ^(2)^	*p* ^(1)^
**Disease severity**					
Moderate	88	3.04	5.01	77.89	0.004 *
Severe	88	4.99	6.26	99.11	
**Outcome**					
Discharge	155	3.96	5.38	82.95	<0.001 *
Death	21	6.27	6.78	129.45	

^(1)^ Mann-Whitney’s test; * statistically significant (*p* < 0.05). ^(2)^ This test groups the two samples by ordering them in an increasing manner (RANK), regardless of the origin of the sample.

## Data Availability

The data that support the findings of this study are available on request from the corresponding authors.
